# The Effectiveness of Art Therapy for Anxiety in Adult Women: A Randomized Controlled Trial

**DOI:** 10.3389/fpsyg.2019.01203

**Published:** 2019-05-29

**Authors:** Annemarie Abbing, Erik W. Baars, Leo de Sonneville, Anne S. Ponstein, Hanna Swaab

**Affiliations:** ^1^Faculty of Health, University of Applied Sciences Leiden, Leiden, Netherlands; ^2^Clinical Neurodevelopmental Sciences, Faculty of Social Sciences, Leiden University, Leiden, Netherlands; ^3^Leiden Institute of Brain and Cognition, Leiden, Netherlands

**Keywords:** anxiety, anxiety disorders, art therapy, emotion regulation, generalized anxiety disorder, social anxiety disorder, panic disorder, social phobia

## Abstract

**Objectives:**

Art therapy (AT) as a treatment option for anxiety is regularly employed in clinical practice, but scientific evidence for its effectiveness is lacking, since this intervention has hardly been studied. The aim was to study the effectiveness of AT on anxiety in adult women. The specific type of AT studied was anthroposophic AT.

**Methods:**

A RCT comparing AT versus a waiting list (WL) condition on anxiety symptom severity, quality of life, and emotion regulation. Factors influencing treatment outcome were additionally explored. Participants were women, aged 18–65 years, diagnosed with generalized anxiety disorder, social anxiety disorder or panic disorder, with moderate to severe anxiety symptoms. The trial was registered in the Dutch Trial Registration (NTR28143).

**Results:**

Fifty-nine women were included, of which 47 completed the trial. Both per-protocol and intention-to treat analyses demonstrated effectiveness of AT compared to WL, showing a reduction in anxiety, an increase in subjective quality of life (both with large effects) and an improvement in accessibility of emotion regulation strategies (medium effect). Treatment effects remained after 3 months follow-up. Improved acceptance of emotions and improved goal-oriented action are aspects of emotion regulation that are associated with the decrease in anxiety level.

**Conclusion:**

AT is effective in reducing anxiety symptoms, improving quality of life and aspects of emotion regulation. Future RCTs should use active controls (treatment as usual) and study cost-effectiveness.

**FIGURE 1:**
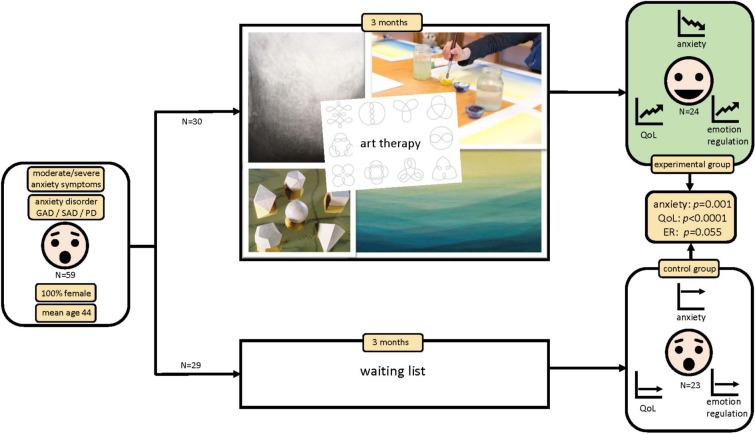
The effectiveness of art therapy in the treatment of anxiety in adult women: a randomized controlled trial.

## Introduction

### Background

#### Anxiety

Nearly 29% of the population will be affected by an AD somewhere in life (Kessler et al., 2005). It is estimated that currently 264 million people live with ADs, and this number increased between 2005 and 2015 with 14,9% (World Health Organization, 2017).

The presence of an AD is associated with a lower quality of life (QoL) and a negative impact on psychosocial functioning (Mendlowicz and Stein, 2000; Cramer et al., 2005). The most common ADs that have an impact on daily life are social anxiety disorder (SAD), generalized anxiety disorder (GAD), and panic disorder (PD) (Anxiety and Depression Association of America [ADAA], 2018). Cognitive behavioral (CBT) and pharmacological therapy (PT) proved to be effective methods for reducing anxiety symptoms (e.g., Kjernisted and Bleau, 2004; Pohl et al., 2005; Hooke and Page, 2006; Hofmann and Smits, 2008). However, ADs have a recurrence rate of 54,8% within 4 years, diagnostically instable recurrences included (Scholten et al., 2016) and a substantial portion of individuals does not benefit from these standard treatments. Not only does PT cause side effects, but also between 20 and 50% of patients have either a contra-indication or don’t respond to PT (Lydiard et al., 1996; Davidson et al., 2004; Blanco et al., 2010; Hyman, 2010). Combination of PT with CBT is recommended (Bandelow et al., 2012) but around 50% of individuals with ADs do not benefit from CBT (Nielsen et al., 2018), or prefer not to take medication,or prefer non-verbal therapy (Uttley et al., 2015). These groups of individuals may benefit from AT.

#### Art Therapy

Art therapy is a non-verbal, experience-oriented therapy that uses the visual arts (e.g., painting, drawing, sculpting, clay modeling) and is provided as standalone therapy or in multidisciplinary treatment programs for anxiety. The non-verbal AT approach is considered to be suitable for individuals with of anxiety, especially if they have difficulty in cognitive (re)labeling of their feelings, or if they are very focused on cognitive labeling and use rationalizing as a psychological coping mechanism (Gold et al., 2004; Smeijsters, 2008). Moreover, the non-verbal AT approach is considered to be suitable for patients with high levels of anxiety, since talking about anxiety and traumas can evoke fear and associated physical reactions (Posthuma, 2001). It is stated that distance to the anxiety can be provided when creating visual art work. To ‘distance’ oneself from the emotion during the act of creating art is believed to improve cognitive regulation of emotions (Smeijsters, 2008). The supposed mechanism is that during the process of creating an art work, one can experience a feeling of being ‘in control,’ which helps to counterbalance the overwhelming experience of anxiety (Van Gerven, 1996).

The effectiveness of AT on reducing anxiety symptoms in adults has hardly been studied in randomized controlled trials (RCTs). There are some indications for effectiveness in different populations, but most of these studies have considerable methodological flaws leading to high risk of bias and are therefore of low quality (Abbing et al., 2018). There is some evidence for effectiveness of AT for treating pre-exam anxiety in undergraduate students (Sandmire et al., 2012) and pre-release anxiety in male prisoners (Zhan Yu et al., 2016). There are no studies on specific ADs like GAD, SAD, or PD (Abbing et al., 2018).

Art therapy has a variety of subtypes, that are based on various perspectives from psychoanalysis, cognitive-analytic therapies, compassion-focused therapy, attachment-based psychotherapy and client-centered approaches, like mindfulness and mentalization-based treatments (British Association of Art Therapists [BAAT], 2018). One of the AT variants with a client-centered approach and with similarities to mindfulness-based treatments is AAT.

An expressive approach is common in most AT interventions (British Association of Art Therapists [BAAT], 2019), in which the client is guided to express feelings, thoughts and life experiences. This approach is also used in AAT, but is combined with an ‘inwardly oriented’ approach, where the therapist offers specific artistic exercises that are often structured and aim to provide ‘impressions’: profound experiences of color and shape. These are thought to activate and strengthen the self-regulating ability of the client.

An important feature of anxiety is the exaggerated cognitive appraisal that is associated with the threatening situation: hyper-alert cognitive schemes lead to pathological anxiety (Beck and Haigh, 2014). The rationale behind AAT is that excessive talking about the anxiety is avoided, to enable patients to deviate from ‘the thinking-mode’ into a ‘feeling-mode’: the aim is to support the individual to obtain ‘profound connection to embodied experiences’: to become aware of the anxiety feelings and responses in the body and learn to influence (downregulate) these feelings, by practicing and experiencing. These processes are thought to be supported through various artistic exercises. The effectiveness of AAT and its working mechanisms is, however, hardly studied and there is currently no adequate theoretical background that provides insight in the specific processes that are influenced by the therapy.

#### Emotion Regulation

Individuals with an AD have more difficulty in regulating emotions compared to individuals without anxiety problems (Suveg and Zeman, 2004; Mennin et al., 2005) and are characterized by dysfunctional ER strategies (Cisler et al., 2010; Ziv et al., 2013; Jazaieri et al., 2014; Diefenbach et al., 2016). People with (for example) GAD have developed an increased intensity of emotions, a lack of understanding of emotions, fear for the emotion, and their response to the emotion is inadequate (Mennin et al., 2002, 2004).

Emotion regulation refers to the intrinsic and extrinsic processes that influence the way in which emotions are expressed or suppressed and are given meaning to, conscious as well as unconscious (Gross and Thompson, 2007). Gratz and Roemer (2004) developed a concept of ER, which involves the “awareness and understanding of emotions, acceptance of emotions, ability to control impulsive behaviors and behave in accordance with desired goals when experiencing negative emotions, and the ability to use situationally appropriate ER strategies flexibly” (Gratz and Roemer, 2004, pp. 42–43). ER can be improved through training and therapy (Baumeister et al., 2006; Tang and Posner, 2009). Artistic exercises, like for example expressive writing, can downregulate emotional distress and promote self-insight (e.g., Pennebaker and Chung, 2007), Thus, ER is an important factor in evaluating AT treatment effects. The connection between AT and ER has already been studied and preliminary established in a narrative review on effectiveness studies (Gruber and Oepen, 2018), primarily focusing on changes in mood in healthy subjects. To gain more insight in the working mechanism(s) of AT on anxiety, it is important to not only investigate the effectiveness of AT on anxiety symptom severity, but also simultaneously explore the role of ER.

### Rationale and Objectives

#### Rationale

Given the need for evidence-based additional treatments for ADs and the lack of methodologically sound effectiveness studies on AT for these indications (Abbing et al., 2018), we designed and executed a study on the effectiveness of AT in reducing anxiety in adult women.

#### Objectives

The *primary objective* was to assess the effectiveness of AT on anxiety and QoL in women with ADs.

The *secondary objective* was to explore factors influencing treatment outcome.

## Materials and Methods

The CONSORT-NPT statement was used for reporting this trial, which is the extension of the CONSORT for randomized trials assessing non-pharmacologic treatments (NPTs) (Boutron et al., 2017).

### Trial Design

**FIGURE 2:**
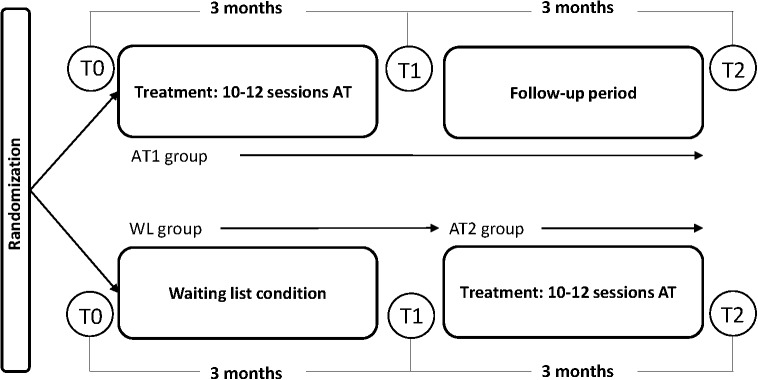
Trial design. AT1 group, art therapy group/experimental group; WL group, waiting list group/control group; AT2 group, second treatment group.

The effectiveness of ATAT on anxiety symptoms in adult women was studied within a RCT. The trial was pragmatic in the sense that it aimed to study the effectiveness of the intervention as it is normally practiced in the field. Participants were pre-stratified on comorbid depression and on psychopharmaceuticals use (see section “Randomization Method and Allocation Concealment”) and subsequently randomly assigned to an experimental group receiving anthroposophic AT (AT1 group) or a control group with participants on a waiting list (WL group), continuing their current treatment, if any, for 3 months. Both groups were measured at baseline (pre-test/T0) and after the intervention/waiting time at 3 months (post-test/T1). The control group then received the intervention (AT2 group) and was assessed immediately after intervention at 3 months. The experimental group was also assessed after 3 months (follow-up/T2) (Figure 2).

Ethical approval was obtained from the Medical Ethical Committee of the Leiden University Medical Centre, Netherlands (NL36861.018.11) and the trial was registered in the Dutch Trial Registration (NTR28143).

### Participants

Participants were recruited through posters/flyers in the practices of family doctors and by social media. Information about the trial was provided through a website where patients could register by filling out a screening instrument [the Dutch version of the Four Dimension Symptoms Questionnaire (4DKL)] (Terluin, 1996; Terluin et al., 2004). Women with moderate to severe anxiety symptoms, scoring >7 for anxiety and/or >10 for distress on the 4DKL (Terluin and Duijsens, 2002), were contacted by phone for eligibility assessment. The inclusion was dimensional in nature: subjects were included primarily based on the level of anxiety symptoms.

Included were adult woman (18–65 years), with GAD, SAD and/or PD (with or without agoraphobia) (diagnosed by means of the MINI-plus diagnostic interview (Sheehan et al., 1998). Candidates were excluded if they had suffered from psychosis or hallucinations, alcohol or drug addiction, suicidal risk and/or brain pathology. Including only women was a post-recruitment decision, since only one male subject fulfilled the inclusion criteria.

Participants signed the informed consent that was approved by the Medical Ethical Committee.

### Intervention and Procedure

The study took place at 25 private AT practices spread throughout the Netherlands, in the period between January 2017 and March 2018.

After randomization AT-participants received 10–12 individual AT sessions of 45–60 min per session during 3 months. Treatment was provided only by qualified and registered Dutch anthroposophic art therapists, with more than 5 years’ experience in working with adults with anxiety. By only including therapists that fulfill the quality criteria stated by the professional organization, it was assured that the intervention deployed in the study was representative for the general approach in AAT.

The treatment was based on common practice and consensus within the Dutch professional association of anthroposophic art therapists (NVKT): first to third session involve intake and free art work, after which treatment goals are set and a therapy plan is made by the therapist, based on intake and observation of client and art work (Huber et al., 2003). This plan consists of a variety of artistic exercises that could be chosen from a list with treatment goals and AT activities, based on consensus within the professional association (Table 1). No fixed treatment protocol was used since anthroposophic AT is a highly individualized treatment. Instead, the exercises on the list could be chosen and adapted to the individual patient, each session taking into account the patients’ specific context. The overview listed several exercises within three media: drawing, painting and clay work (Table 1). The contents of the therapy processes (treatment goals and exercises) were documented by the therapists. Afterward, researchers checked if the deployed activities fulfilled the list of treatment goals and exercises. The WL participants were on the WL for 3 months and received AT 3 months later (Figure 2).

**Table 1:** List of artistic exercises and therapy goals, approved by the Dutch AAT association.

Session	Aims	Exercises
Session 1–3	Intake Set treatment goals and plan	Free art work
Sessions 4–10	Optional treatment goals: *Creating a feeling of safety Experiencing boundaries Strengthening objectivity Reinforcing connection with feeling* *Promoting relaxation Reinforcing self-confidence*Other treatment goals are allowed; mention these treatment goals here and describe why these are important in the treatment of your client.	Optional artistic exercises: • Clay: *Clay modeling of a sphere.* *Clay modeling of platonic solids.* *Transformation of (symbolic) shapes.* • Drawing: *Expression of fear in free drawing.* *Atmospheric images in relation to inner feeling, with pastel drawing.Shape drawing (loops).* *Drawing from observation.* *Light-dark exercises with charcoal. Color exercises with pastel.* • Painting: *Expression of fear in free painting. Color exercises in wet-on-wet technique (aquarelle paint on wet paper).* Other exercises are allowed; provide rationale for these exercises and a description.
Session 12	Evaluation	

### Measures

The following measures were used for screening, diagnosing and determining anxiety symptom severity, QoL, and ER.

#### Screening for Psychological Problems

Participants were screened by the 4DKL (Terluin, 1996). This is a questionnaire for adolescents and adults and screens on psychological problems with 50 items, measuring symptoms of distress, depression, anxiety, and somatic symptoms. Anxiety symptoms are measured by 12 items. This instrument is reliable and valid (Egberink et al., 2005).

#### Diagnostic Interview for Anxiety Disorders and Comorbidity

Psychopathology was assessed using the Dutch version of the rater-administered Mini International Neuropsychiatric Interview Plus (MINI-Plus) (Van Vliet et al., 2000), which is a comprehensive diagnostic semi-structured interview. In the present study, the MINI-Plus was used to assess the type(s) of AD and the presence of (comorbid) depression, PTSS and substance abuse (exclusion criterium).

#### Primary Outcome: Level and Dimensions of Anxiety

The Dutch version of the Lehrer Woolfolk Anxiety Symptom Questionnaire (LWASQ) (Lehrer and Woolfolk, 1982) was used to measure the anxiety level. The LWASQ is a self-report, generic anxiety instrument with 36 questions which assesses the cognitive (worry and rumination), behavioral (avoidance) and somatic (physical symptoms) aspects of anxiety. The reliability of the LWASQ is sufficient (α = 0.83 to 0.92) and the questionnaire is suitable for the measurement of treatment effects (Scholing and Emmelkamp, 1992).

#### Secondary Outcomes: Subjective Quality of Life and Emotion Regulation

The Dutch version of the MANchester Short Assessment of QoL (MANSA) (Priebe et al., 1999; Van Nieuwenhuizen et al., 2000) was used to measure QoL. This instrument consists of 12 questions that measure the satisfaction with, e.g., life in general, work and friendships. The MANSA is a reliable instrument (Janssen-de Ruiter et al., 2015).

To measure the difficulties that patients experience in ER, the Dutch version of the Difficulties in Emotion Regulation Scale (DERS) (Gratz and Roemer, 2004) was used. The questionnaire consists of 36 items in six domains: (1) lack of clarity of emotions, (2) lack of awareness of emotions, (3) difficulty in controlling impulses, (4) non-acceptance of emotions, (5) limited access to ER strategies, and (6) difficulty with goal-oriented action (Gratz and Roemer, 2004).

The DERS can be reliably deployed and interpreted in different demographic groups (Ritschel et al., 2015). The construct validity and internal consistency (Cronbach’s α > 0.80) is sufficient for all scales (Gratz and Roemer, 2004; Neumann et al., 2010). The test–retest reliability for the total score is good (*r* = 0.88; subscales 0.56 < *r* < 0.90).

#### Procedure of Measurements

All participants completed online assessments of the 4DKL, LWASQ, MANSA, and DERS at three time points (Table 2).

**Table 2:** Procedure.

	Phase I		Phase II
	T0	T1 (+3 months)	T2 (+3 months)
AT1	Pre-treatment	Post-treatment	Follow-up
WL	Pre-wait time	Post wait time/Pre-treatment AT2	Post-treatment AT2

All questionnaires were administered with Qualtrics Survey Software (Qualtrics Software (2005), Provo, UT, United States, version 2017).

### Sample Size

Sample size calculation was based on a pre–post measurement difference in the primary outcome of 15% (as this was considered a clinically relevant decrease in LWASQ total score), with an alpha of 0.05 and a power of 0.80. Considering a dropout rate of 15%, the estimated sample size was 30 patients per group; a total of 60 participants^1^.

### Randomization Method and Allocation Concealment

**FIGURE 3:**
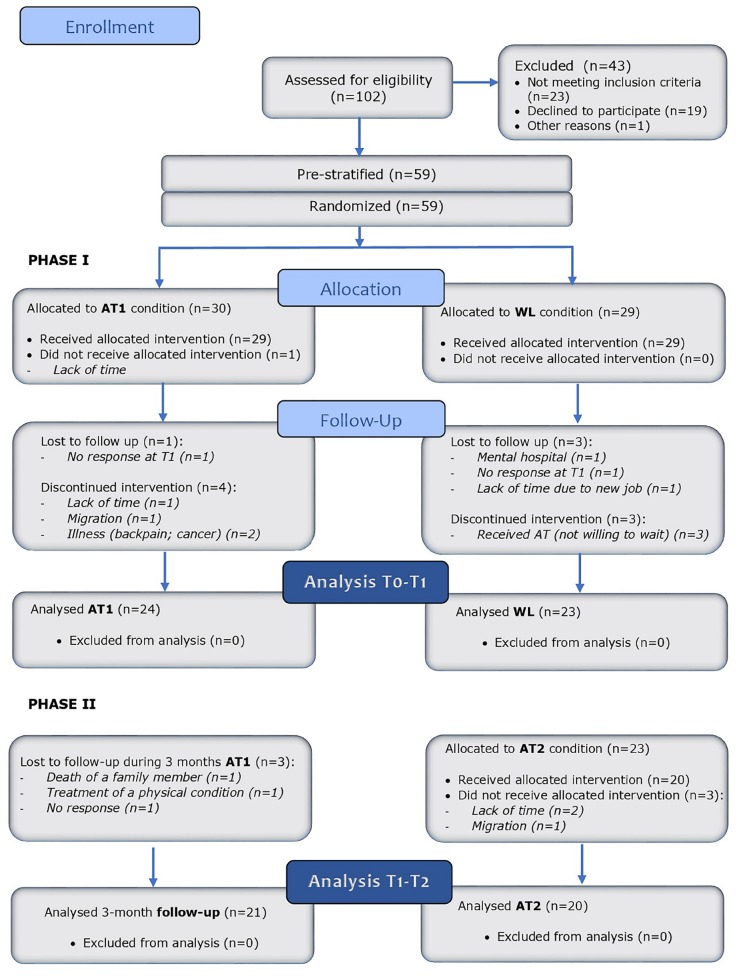
Study flow diagram.

Participants were pre-stratified into four strata: whether or not using psychotropic drugs, and whether or not having moderate or severe depression symptoms (4DKL: depression > 6), and subsequently assigned to treatment (AT) or control group (WL) by means of block randomization (blocks of 2) (Figure 3).

Participants received a participation number at enrolment. After enrolment and stratification of participants by AA, a list with the random allocation sequence was generated by EB through computer selection ^2^. AA assigned participants to intervention according to the randomization list.

Art therapists and participants could not be blinded.

### Statistical Methods

Statistical analyses were conducted using SPSS statistics (version 23.0) (IBM Corp., 2015). All data were checked for normal distribution using the Shapiro–Wilk test, Q–Q plot and histogram.

#### Evaluation of Baseline Differences

The randomization was evaluated by comparing experimental and control group at baseline. For normal distributed, continuous variables an independent *t*-test was used and the variables were presented as mean ± standard deviation (SD). For categorical variables Pearson’s chi-squared test was applied and variables were presented as number and/or percentage.

#### Missing Values

Reasons for missing values were reported. Dropouts were compared to completers using pre-test measures on age, anxiety score, depression score and QoL score, by use of independent students *t*-tests. If no significant differences were found, the missing cases were deleted and per protocol (PP) analyses were performed for all outcomes.

For the intention-to-treat (ITT) analysis missing values on anxiety score at baseline (T0) and T1 for all participants that were randomized to one of the two groups were imputed based on two theoretical models (White et al., 2011). In the first model participants with no measurements at T0 and T1 received anxiety scores that were the mean of the condition they were allocated to and at T1 the same score was imputed [last observation carried forward (LOCF) procedure], expressing that there was no treatment effect and participants were comparable to the average participant. Participants with no measurement at T1 received the anxiety score at T0 (LOCF procedure). The second model was the same, with the only difference that the participants with no measurements at T0 and T1 received anxiety scores that were the highest possible score, expressing the worst-case scenario that these participants were the ones with highest anxiety level.

#### Hypotheses

The following hypotheses were tested: (1) AT is superior to WL in reducing anxiety symptoms and improving QoL in adult women with ADs; (2) the effects of AT remain at 3 months follow-up; and (3) the effects of AT are confirmed in the WL group that receives AT 3 months later.

#### Evaluation of Treatment Effects

To examine hypothesis 1, a general linear model repeated measures analysis for variance (RM-ANOVA) was used, using outcomes of LWASQ at pre- and post-treatment as levels of the within-subject (WS) factor Test moment (T0 vs. T1) and Group (AT1 vs. WL) as between-subjects (BS) factor. To conclude that the treatment has a positive effect, the Test moment^∗^Group interaction must be significant and in the right direction. To test if reduction of anxiety was different for the three subscales of the LWASQ, these subscales were added as levels of the WS factor Scale in a second analysis.

Likewise, for the secondary outcomes (MANSA, DERS), RM-ANOVAs were performed. If trend significant interactions were found, further explorative analyses (paired *t*-tests) were executed to measure within-group effects.

A PP analysis was performed for all primary and secondary outcome variables. In addition, an ITT analysis was performed for the primary outcome variable ‘level of anxiety.’

A *p*-value of 0.05 was considered statistically significant. The effect size partial eta squared ($\eta_{p}^{2}$) was calculated to assess the magnitude of the effect. An effect size of 0.01–0.06 is considered a small effect, 0.06–0.14 a medium, and >0.14 a large effect in RM analysis (Borenstein, 2009).

For hypothesis 2, a RM-ANOVA with Test moment (T0, T1, T2) as WS factor on the primary and secondary outcomes of the AT1 group was performed, to determine if treatment effects remain for (at least) 3 months and to test if an effect (compared to baseline) still exists, using a simple contrast with T0 as the reference level (T0 vs. T1, T0 vs. T2).

Hypothesis 3 was tested with paired *t*-tests on T1–T2 outcomes (pre- vs. post-treatment) of the WL group that received treatment (AT2).

#### Exploration of Factors Influencing Anxiety Reduction

To explore factors that influence anxiety symptom reduction, correlations were computed between the primary outcome variable (pre–post treatment difference in anxiety symptom severity, PP analysis) and the pre–post treatment difference scores on QoL, distress, somatization and difficulties in ER., Only the significant correlations were further studied with regression analysis within the total treatment group (AT1 and AT2 together), to examine if improvements of ER were associated with anxiety symptom reduction. An ANCOVA with pre- and post-treatment scores of anxiety, and the pre–post treatment difference score of ER as covariate was performed as *post hoc* analysis.

To explore pre-treatment factors that would favorably affect the success of treatment, the same procedure was followed, but with pre-treatment measures of age, duration of anxiety, comorbidity (number) and level of education. Regression analyses were conducted within the total treatment group (AT1 and AT2 together), with the primary outcome variable (pre–post treatment difference in anxiety symptom severity, PP analysis) and the pre-treatment measures that showed a significant correlation with the anxiety difference score.

## Results

### Participant Flow

In the period January 2017 until July 2017, 102 persons applied for the trial and were screened for eligibility. A total of 43 patients was excluded for not meeting the inclusion criteria (*n* = 23) or not willing to participate (*n* = 19) or for other reasons (*n* = 1).

In total, 59 participants were included and randomized after stratification. The distribution over the four strata was as follows: no depression and no psychopharmaceuticals (*n* = 27), no depression and psychopharmaceuticals (*n* = 11), depression and no psychopharmaceuticals (*n* = 14), depression and psychopharmaceuticals (*n* = 7).

Thirty participants were assigned to the intervention group (AT1) and 29 to the control group (WL). During the study, 12 participants dropped out, six from the AT1 group and six from the WL group. Loss-to-follow-up occurred in the AT1 group (*n* = 1), as well in the WL group (*n* = 3). In total, data of 47 participants were analyzed in the PP analysis: 24 in the AT1 group and 23 in the WL group.

Participants of the AT1 group were followed-up around 3 months after completion of the treatment. Participants of the WL group received AT (AT2 group) after completion of the 3 months wait time. Three participants of AT1 group were lost to follow-up and three participants of the AT2 group did not receive the intervention for several reasons (Figure 3).

### Missing Values

Twelve participants (20%) dropped out and 47 participants (80%) completed Phase I of the trial. There were no significant differences between dropouts and completers on baseline parameters: age, anxiety score (LWASQ), anxiety score (4DKL), depression score (4DKL), distress score (4DKL), somatization score (4DKL), and QoL (MANSA) (0.87 < *p* < 0.29). These results indicate that missings were completely at random and could be list-wise deleted, without risk of bias, and further analyses are per-protocol.

### Baseline Characteristics

Table 3 gives an overview of the baseline characteristics of participants. The participants did not differ on key variables, including age, diagnosis, use of medication, occupation, education, familiarity with AM and outcome variables at baseline.

**Table 3:** Participants’ baseline characteristics.

Characteristics		AT (*n* = 24) *Mean (SD) or n (%)*	WL (*n* = 23) *Mean (SD) or n (%)*	*p*
Age (years)		42.4 (14)	46.5 (14)	0.32
4DKL anxiety	>7 moderate; >12 severe	12.0 (4.3)	10.3 (4.9)	0.21
Anxiety score	LWASQ total score	103.2 (21.4)	97.2 (21.7)	0.34
Anxiety disorder according to MINI+	GAD	14 (58%)	11 (48%)	0.47
	SAD	9 (38%)	12 (52%)	0.31
	PD	14 (58%)	14 (61%)	0.61
	Comorbid PTSD	4 (17%)	6 (26%)	0.40
Duration	Of anxiety symptoms (years)	15.1 (±18.6)	20.2 (±19.4)	0.34
4DKL distress	>20 severe	23.0 (6.2)	22.6 (5.0)	0.81
4DKL depression		4,0 (2,7)	4,2 (3.3)	0.77
Depression according to MINI+	Current depression	3 (13%)	2 (9%)	0.46
	Previous depression	7 (29%)	9 (39%)
Medication		7 (29%)	8 (35%)	0.51
Other therapies during study period		6 (25%)	5 (22%)	0.41
Occupation	Working	6 (25%)	7 (30%)	0.52
	Self employed	3 (13%)	3 (13%)	
	Student	2 (8%)	2 (9%)	
	Unemployed	3 (13%)	2 (9%)	
	Sick leave/incapacitated	9 (38%)	9 (39%)	
Education	Low	3 (13%)	2 (9%)	0.14
	Moderate	9 (38%)	4 (17%)	
	High	11 (46%)	17 (74%)	

*4DKL, Four-Dimensional Symptom Questionnaire; AT, art therapy group; WL, waiting list group; Education, Low (elementary school, vmbo, mbo1), Moderate (havo, vwo, mbo2-4), High (HBO, WO); Medication, psychotropic medication; p-values < 0,05 were considered significant.*

The analyzed sample of 47 participants had a mean age of 44.4 years (*SD* = 14.0), moderate to severe anxiety symptoms: 11.2 (*SD* = 4.6), a mean duration of anxiety symptoms of 17.6 years (*SD* = 18.9) [range: 3 months – 64 years (lifetime)]. Medication for anxiety was used by 15 participants and 11 participants received other therapies next to AT (psychotherapy, EMDR, and acupuncture).

Multiple anxiety diagnoses applied to all participants (comorbid ADs), with two to five ADs per person. The criteria for the diagnosis GAD were met 25 times, for SAD 21 and for PD 28 times. Ten participants suffered from (comorbid) PTSD, five participants had current comorbid depression and 16 participants experienced one or more depressive episodes prior to this study.

### Features of the Experimental Treatment

In total, 44 participants completed the therapy, and 37 case files were received until September 2018 and analyzed. All cases fulfilled the criteria of an AAT intervention as described in the study protocol: use of anthroposophic AT exercises from the predefined list and adaptation of the intervention to the specific context of each individual patient (optional).

Therapy plans consisted of artistic exercises in which the media drawing and clay modeling were used most often, respectively in 37 and 34 of the analyzed 37 cases, and painting in 21 cases. Drawing exercises consisted of shape drawing, charcoal drawing, pastel drawing, and visualization exercises. The most deployed techniques were shape drawing (drawing of relaxing loops), often provided as homework exercise, the creation of light-dark contrasts and conversions (charcoal drawing), drawing from observation and working on atmospheric images in relation to inner feeling (pastel drawing). The expression of the fear, with various materials and techniques, often preceded by a visualization exercise, was also used in most cases. Within the clay medium, round shapes were most frequently used, as well as the modeling of one or more platonic solids. Transformation processes and symbolic exercises were also frequently applied within the clay medium. Painting exercises were mainly the wet-in-wet technique (aquarelle paint on wet paper) and were mainly used in the first sessions as free art work. For examples, see Figure 1 (visual abstract).

### Treatment Effects – Primary Outcomes

#### Per Protocol Analysis

**FIGURE 4:**
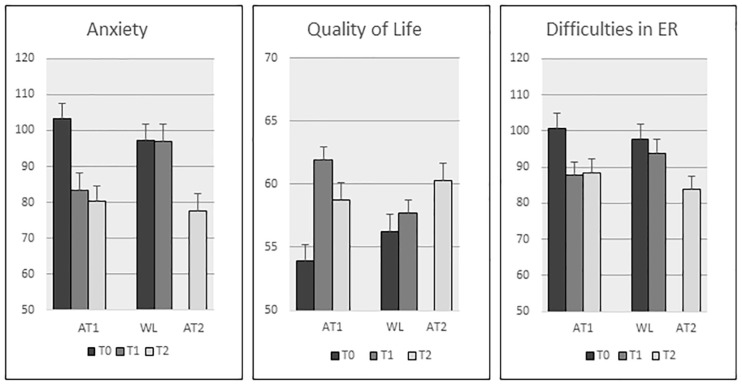
Primary and secondary outcomes at T0, T1, and T2. *Means* (*SE*).

On the primary outcome anxiety symptom severity, the interaction effect Test moment^∗^Group was significant: *F*(1,45) = 11.49, *p* = 0.001, with a large effect size ($\eta_{p}^{2}$= 0.20), showing that anxiety was reduced in the AT1 group but not in the WL group (see Figure 4). The three subscales of the LWASQ, added in a second analysis as levels of the WS factor Scale, showed no significant interaction Test moment^∗^Group^∗^Scale (*p* = 0.71), reflecting that the improvements in anxiety symptom severity hold equally for the somatic, behavioral, and the cognitive area.

The within-group outcomes (mean differences, SDs, 95% CIs and *p*-values) are presented in Supplementary Table 1.

#### Intention to Treat Analysis

Both ITT analyses for the primary outcome (LWASQ) demonstrate the same significant differences between the groups: *p* = 0.011 with a medium effect size ($\eta_{p}^{2}$ = 0.11).

### Treatment Effects – Secondary Outcomes

#### Quality of Life

The interaction Test moment^∗^Group was *F*(1,45) = 22.94, *p* < 0.0001 and the effect size was large ($\eta_{p}^{2}$ = 0.52), reflecting, that QoL was increased in the AT group but not in the WL group (Figure 4).

#### Emotion Regulation

The interaction Test moment^∗^Group was trend significant for difficulties in ER (total score): *F*(1,45) = 3.87, *p* = 0.055, and was accompanied by a medium effect size ($\eta_{p}^{2}$ = 0.08). *Post hoc* analysis confirmed that total ER improvement was significant in the AT group (*p* = 0.003) but not in the WL group (*p* = 0.16). On the subscale level, the only significant interaction Test moment^∗^Group was on the subscale *limited access to ER strategies*: *F*(1,45) = 6.0, *p* = 0.018, $\eta_{p}^{2}$ = 0.12. This indicates that participants had better accessibility to ER strategies after therapy.

The subscales *lack of clarity of emotions*, *non-acceptance of emotions,* and *limited access to ER strategies* showed significant improvements in the AT group (within-group analysis) (0.008 < *p* < 0.05) (Table 3).

### Follow-Up Outcomes in First Treatment Condition

The first treatment group (AT1) was followed up 3 months after treatment (*n* = 21). Using a simple contrast with T0 as reference level (first contrast: T0 vs. T1, second contrast: T0 vs. T2), the RM-ANOVA on anxiety symptom severity revealed a significant first and second contrast [*F*_T0vs.T1_(1,20) = 10,68, *p* = 0.004, $\eta_{p}^{2}$= 0.35; *F*_T0vs.T2_(1,20) = 16.51, *p* = 0.001, $\eta_{p}^{2}$= 0.45]. Similar effects were observed for QoL [*F*_T0vs.T1_(1,20) = 41.1, *p* < 0.0001, $\eta_{p}^{2}$= 0.67, *F*_T0vs.T2_(1,20) = 12.56, *p* = 0.002, $\eta_{p}^{2}$= 0.39] and ER [*F*_T0vs.T1_(1,20) = 9.04, *p* = 0.007, $\eta_{p}^{2}$= 0.31, *F*_T0vs.T2_(1,20) = 14.43, *p* = 0.001, $\eta_{p}^{2}$= 0.42]. The observed treatment effects on anxiety symptom severity, QoL and ER remained at follow-up (Figure 4). The outcomes at T2 were still significantly improved compared to baseline (T0).

### Outcomes in Second Treatment Condition

The second treatment group (AT2, *n* = 20), showed similar improvements as the first treatment group: anxiety is significantly lowered in the AT2 condition [mean(SD)]: 95(24,10)–77,55(21,57), *p* = 0.001, with a large effect size ($\eta_{p}^{2}$= 0.45).

However, there are some differences. The improvement in QoL was not significant in the AT2 condition [mean(SD)]: 58,05(6,93)–60,30(9,13), *p* = 0.11 ($\eta_{p}^{2}$= 0.13), while it was highly significant in the AT1 group. The improvement in total ER was significant in de AT2 condition [mean(SD)]: 94,45(19,83)–83,95(21,59), *p* = 0.003, ($\eta_{p}^{2}$= 0.38), and associated with a larger effect size compared to the AT1 group.

### Exploration of Factors That Influence Anxiety Reduction

The total treatment group (*n* = 44) consisted of AT1 (*n* = 24) and AT2 (*n* = 20). The mean difference in anxiety symptom severity of the total treatment group was 18,68 (*SD* = 21,96) (95% CI: 12,01–25,36, *p* < 0.0001), which represents an anxiety severity decrease of 18,6%.

#### Role of Emotion Regulation in Anxiety Reduction

The LWASQ difference score (pre–post treatment) was correlated with the difference scores of the other outcomes (MANSA, DERS). ER difference score was correlated with anxiety symptoms (*r* = 0.39, *p* < 0.0001), reflecting that a decrease in anxiety symptoms was associated with an increase in ER. Looking at the subscale level, improvement on five of the six ER subscales was associated with a decrease of anxiety symptoms: *clarity of emotions* (*r* = 0.30, *p* = 0.005), *controlling impulses* (*r* = 0.24, *p* = 0.024), *acceptance of emotions* (*r* = 0.43, *p* < 0.0001), *access to ER strategies* (*r* = 0.27, *p* = 0.013) and *goal-oriented action* (*r* = 0.31, *p* = 0.004).

An explorative backward regression analysis with these variables resulted in a significant model [*F*(2,41) = 17.55, *p* < 0.0001, *R*^2^= 0.461]. The model consisted of two subscales of the DERS: improvement in *Non-acceptance of emotions* (β = 0.556, *t* = 4,39, *p* < 0.0001) and improvement in *Difficulties with goal-oriented actions* (β = 0.220, *t* = 1,739, *p* = 0.09) explaining 46,1% of the variance in anxiety symptom reduction.

The *post hoc* ANCOVA on anxiety level showed a significant interaction [*F*(1,22) = 29,52, *p* < 0.0001, $\eta_{p}^{2}$ = 0.57] between Test moment (pre vs. post treatment) and the covariate reduction of difficulties in ER, reflecting that larger anxiety reduction was highly associated with larger improvement in ER.

#### Baseline Factors That Influence the Success of Treatment

The LWASQ difference score (pre–post treatment) was correlated with age, duration of anxiety, number of comorbidities, education, familiarity with AT or anthroposophic healthcare, pre-treatment levels of anxiety (LWASQ), QoL (MANSA), ER (DERS), distress, depression and somatization (4DKL). Only pre-treatment levels of anxiety (*r* = 0.38, *p* < 0.0001) and ER (*r* = 0.25, *p* = 0,017) showed a significant correlation with therapy success (anxiety reduction).

A regression analysis (Method = Enter) resulted in a significant model [*F*(2,41) = 6.30, *p* = 0.004, *R*^2^= 0.235] with pre-treatment level of anxiety (β = −0.350; *t* = −2.33) and pre-treatment ER score (β = −0.220; *t* = −1.46), together explaining 23,5% of the variance in anxiety symptom reduction.

## Discussion

### Summary of Outcomes

This study is the first RCT that studied an AT intervention for GAD, SAD, and PD. For this reason and because anthroposophic AT as a complex intervention is adjusted to the needs of individual patients, the RCT had a pragmatic character. To evaluate the intervention as provided in clinical practice, therapists were allowed to deploy the treatment as they would normally do. The tested intervention was executed by trained AAT professionals who are able to individualize the treatment within the boundaries of the described goals, means and exercises, based on consensus within the professional organization. Artistic exercises with clay, drawing, and painting were used in every case to work on anxiety reduction. The most used medium was drawing in particular shape drawing, often as ‘warming-up’ and also as homework exercises. The second most used medium was clay work): the modeling of a sphere or other round shapes and metamorphosis series of platonic solids were the most frequent deployed exercises.

The outcomes show that 10–12 sessions of AT lead to a significant decrease of anxiety symptoms, as well as a significant improvement in QoL and remained at 3 months follow-up. Significant improvements were also observed with respect to *access to emotion regulation strategies*. Improvements in ER were highly associated with anxiety reduction: the ER aspects *acceptance of emotions* and *improved goal-oriented action* accounted for 46% of the improvement in anxiety symptom severity. Participants with higher pre-treatment anxiety scores and those who experienced many difficulties in ER pre-treatment showed the largest improvements.

### Interpretation and Comparison to Literature

Effects of AT on anxiety in adults have been suggested in other studies (Sandmire et al., 2012; Moayer Toroghi, 2015; Zhan Yu et al., 2016), although these studies have methodological issues resulting in a high risk of bias (Abbing et al., 2018) and do not concern subjects with specific or diagnosed ADs.

Anxiety symptoms are related to less effective ER (Suveg and Zeman, 2004; Mennin et al., 2005). The association of improved ER with anxiety reduction in our study is in line with results from various studies which show that a decrease of anxiety is related to improvement of ER (Cisler and Olatnuji, 2012). Our RCT showed that ER is a factor that influences anxiety reduction through AT. The improvements in ER that had the largest influence were o better *acceptance of emotions* and improved *goal-oriented action*. Usually, ER training focuses on strategies that minimize negative emotions and/or maximize positive emotions (Koole and Aldao, 2016). These strategies largely fall within need-oriented ER and appear to provide limited contributions to psychological health (Aldao and Nolen-Hoeksema, 2012). Instead, Koole and Aldao (2016) argue that the focus should be more toward goal-oriented ER and person-oriented ER, to learn to apply strategies more flexibly and adaptively. The improvement of *goal-oriented action* in our study suggests that AT promotes goal-oriented ER. AT may also improve person-oriented ER, since to gain *acceptance of emotions*, it is needed to face the emotion and to endure this. This could be easier and less threatening if the emotion can be faced in externalized form, which is the case in art work (Haeyen, 2018).

Higher pre-treatment scores of anxiety were predictive of therapy success. This seems plausible, because the higher the score, the more room for improvement. Another possible explanation is that AT is most suitable for patients with severe anxiety symptoms.

### Strengths and Limitations

The strength of this study is the RCT design, being used to study the effectiveness of AT in reducing anxiety in subjects with ADs for the first time. Other strengths are the broad inclusion criteria that were used, to assess whether the intervention could be helpful to most women with moderate to high levels of anxiety symptoms (dimensional approach), and not just to a narrow diagnostic subgroup.

The set of relevant outcome variables (anxiety symptom level, QoL, and ER) enabled us to explore a possible working mechanism. The strength of the LWASQ is that it is able to measure both cognitive, behavioral, as well as somatic aspects of anxiety. This enabled us to investigate in what area of anxiety the improvements occurred. A limitation is that this instrument is not as commonly used in effectiveness studies on anxiety as the State-Trait Anxiety Inventory (STAI) (Spielberger et al., 1983). Our outcomes are therefore not easy to compare to (anxiety) outcomes of other studies.

A more important limitation is the risk on performance bias as blinding is not feasible in AT, like in other psychotherapeutic interventions. According to Munder and Barth (2017), the risk can be lowered by using an active treatment as control. We used a waitlisted (inactive) control group, which is the most logical first step in this young research domain. A placebo effect may therefore have biased the results in that the effect of treatment may be overestimated. Important aspects that may have influenced the observed effectiveness are expectations and motivation of the participants. Positive expectations lead to more positive self-evaluation. It is likely that the study population consisted of women who have (at least some) affinity with creativity and/or art making, because the participants applied for this trial themselves. This resulted in a study population that might have had positive expectations of the therapy. It is estimated that a positive expectation causes 15% of the effects of psychotherapy (Asay and Lambert, 1999), because these expectations can lead to a more positive self-evaluation of mental health (Taylor and Brown, 1988). Motivation is also known to be an important factor in therapy success (Gordon, 1976; Hubble et al., 1999) and contributes to the improvement of general health and wellbeing (e.g., Deci et al., 1991; Miller et al., 1993; Pelletier et al., 1997). Thus, the therapeutic effect of AT may be somewhat overestimated in our study. In psychotherapy it is argued that ‘treatment’ leads to better outcomes than ‘no treatment,’ due to non-specific treatment factors (e.g., empathy, warmth, attention) (Wampold, 2001; Bjornsson, 2011). It might therefore be obvious that the AT participants improved compared to the WL participants. However, based on the work by Kiene (2013), there are some arguments that support the hypothesis that the observed effects are not only caused by non-specific treatment factors, but can (partly) be attributed to the specific effect of AT: the effect size is large, the effect occurs relatively fast (within 3 months, compared to the mean duration of anxiety of 17.6 years), the effects remain at follow-up (3 months), the effects were repeated in the second treatment group (the previous WL group), and there is evidence of a rational working mechanism (AT contributes to better *acceptance of emotions* and improved *goal-oriented action*, leading to improvement of ER skills) that is in line with AT expertise and literature. Another important limitation is that our study does not provide insight in the specific AT factors that contributed to the observed effects. Our study provides some information about the content of the intervention, but treatment goals from the list are not connected to general accepted theories and a rationale for the deployed artistic exercises cannot be provided at this point. This should be subject of future studies, aimed at further opening-up the black-box of AT. A final limitation is that we were not able to perform subgroup analyses per subtype of anxiety, due to small subgroups and overlap in diagnostic groups, since multiple diagnosis applied to all subjects.

### Generalizability

The study of a complex intervention, utilizing customized care within a range set by professionals, strengthens the external validity of the results. The study population consisted mainly of moderate to highly educated women, with multiple anxiety diagnoses, moderate to severe anxiety symptoms and a long duration of symptoms. Individuals with high levels of anxiety, comorbidity and a long duration of symptoms generally have low therapy success rates (e.g., Mululo et al., 2012). The outcomes of our study indicate that this complex population benefited from AT, indicating that AT can be an option for this specific group of patients, and might also be beneficial for less complex anxiety patient groups. Anthroposophic AT is a treatment that is tailored to the individual, with could partially explain the positive results: patients with severe symptoms, comorbidities and a chronic course appear to be better treatable with a therapy that is adapted to the individual, in terms of intensity and focus (Newman et al., 2013).

Participants applied for this trial themselves. It is therefore likely that the study population consisted of women who have (at least some) affinity with creativity and/or art making and were motivated to try this therapy. It is not clear if the results are generalizable to less motivated women with anxiety. Since only women were included, the results are not generalizable to men.

The tested AT intervention was only executed by trained AAT professionals who are able to individualize the treatment within the boundaries of the described goals, means and exercises, based on consensus within the professional organization. Therefore the tested intervention is representative of for the AAT treatment of anxiety. Based on the encouraging results of our study, AT as an optional treatment for anxiety can be continued in clinical practice.

### Future Perspectives

Further studies are needed to strengthen the evidence base for AT in the treatment of anxiety. Studies with active controls are recommended, since this reduces the risk of bias due to the lack of blinding. A sham treatment could correct for the effect of ‘being treated.’ AT should also be compared to treatment as usual (e.g., CBT). To assess the long-term effects of AT, longer follow-up periods (>6 months) are needed.

In future RCTs, the inclusion criteria may be narrowed down to further explore the effects of AT on specific ADs. The use of more objective measures, like physiological measures of anxiety, in addition to the present measures are also recommended. In future studies executive functioning may be included to further unravel the working mechanisms of AT as it is known that EF is negatively influenced by anxiety (Fujii et al., 2013).

Further studies aimed at the therapeutic content of AT are needed to provide insight into AT-specific factors that contribute to the observed effects. AAT could also be compared to other types of AT. Studying the cost-effectiveness of AT compared to treatment as usual (CBT, pharmacotherapy, or a combination of both), is important to ascertain the contribution of AT to value-based healthcare. Finally, client experiences, obtained through in-depth interviews, can give additionally insight in the subjective value of this treatment for patients and the specific treatment factors that contribute to the reduction of anxiety symptoms and the improvement of QoL.

## Conclusion

(1) Three months (10–12 sessions) AT is superior to WL condition in reducing anxiety symptoms and improving QoL in adult women with ADs GAD, SAD and/or PD and moderate to severe anxiety symptoms. These effects remain at 3 months follow-up.
(2) Positive changes in ER, especially in the acceptance of emotions and in improved goal-oriented action, account for 46% of the reduction of anxiety symptom severity.
(3) To obtain high quality evidence for effectiveness of AT, RCTs with active controls (treatment as usual) and RCTs on cost-effectiveness are needed.

## Ethics Statement

This study was carried out in accordance with the recommendations of the Medical Ethics Committee of the Leiden University Medical Centre with written informed consent from all subjects, in accordance with the Declaration of Helsinki. The protocol was approved by the Medical Ethics Committee of the Leiden University Medical Centre, the Netherlands (NL36861.018.11).

## Author Contributions

AA was the principal investigator, coordinator, and executive researcher of the trial. AP assisted with the screening of participants and collecting the case files. Statistical advice was provided by LdS and EB, who was also the consulting researcher. Overall responsibility lies with HS as the head of the research team. All researchers provided input to the article.

## Conflict of Interest Statement

The authors declare that the research was conducted in the absence of any commercial or financial relationships that could be construed as a potential conflict of interest.

## Footnotes

1 http://clincalc.com/stats/samplesize.aspx

2 www.randomization.com

## Abbreviations

AAT: anthroposophic art therapy
AD: anxiety disorder
AT: art therapy
ER: emotion regulation
WL: waiting list.
